# Successful treatment of metastatic uveal melanoma with ipilimumab and nivolumab after severe progression under tebentafusp: a case report

**DOI:** 10.3389/fonc.2023.1167791

**Published:** 2023-05-03

**Authors:** Selina Reiter, Christopher Schroeder, Julian Broche, Tobias Sinnberg, Irina Bonzheim, Daniela Süsskind, Lukas Flatz, Andrea Forschner

**Affiliations:** ^1^ Department of Dermatology, University Hospital of Tübingen, Tübingen, Germany; ^2^ Institute of Medical Genetics and Applied Genomics, University Hospital of Tübingen, Tübingen, Germany; ^3^ Institute of Pathology and Neuropathology, University Hospital of Tübingen, Tübingen, Germany; ^4^ Department of Ophthalmology, University Hospital of Tübingen, Tübingen, Germany

**Keywords:** tebentafusp, immune checkpoint inhibitors, uveal melanoma, bi-specific therapy, ctDNA

## Abstract

Metastatic uveal melanoma (UM) is a rare form of melanoma differing from cutaneous melanoma by etiology, prognosis, driver mutations, pattern of metastases and poor response rate to immune checkpoint inhibitors (ICI). Recently, a bispecific gp100 peptide-HLA-directed CD3 T cell engager, tebentafusp, has been approved for the treatment of HLA-A*02:01 metastatic or unresectable UM. While the treatment regime is complex with weekly administrations and close monitoring, the response rate is limited. Only a few data exist on combined ICI in UM after previous progression on tebentafusp. In this case report, we present a patient with metastatic UM who first suffered extensive progression under treatment with tebentafusp but in the following had an excellent response to combined ICI. We discuss possible interactions that could explain responsiveness to ICI after pretreatment with tebentafusp in advanced UM.

## 1 Introduction

Uveal melanoma (UM) is a rare tumor of the eye, most often arising from the melanocytes located in the choroid, with an incidence of about 5 cases per million per year. Studies showed that there is a geographic north-to-south decreasing gradient of incidence, probably due to the lack of protective effect of ocular pigmentation in northern, mostly Caucasian, populations. UM shows a rising incidence in positive correlation to age with a peak at 70 years. No significant difference between male and female is known (1).

Unlike cutaneous melanoma, which usually is associated with lymphatic metastasis but can also spread through blood, UM usually metastasizes only hematogenously. For this reason, the pattern of metastatic spread includes predominantly the liver (89%), but also the lung (29%) and bones (17%). The risk of metastases in uveal melanoma is high, as approximately 50% of patients develop metastases within 10 years after initial diagnosis. Median survival is 6 to 12 months once metastasis occurred (2).

Chromosomal aberrations and gene alterations are often found in metastatic UM and may be associated with distinct prognosis. For example, monosomy of chromosome 3 and chromosome 8 alterations are, especially when occurring simultaneously, associated with a worse prognosis. Furthermore, mutations in *BAP1* (BRCA1 Associated Protein 1) or *SF3B1* (Splicing Factor 3b Subunit 1 gene) are known risk factors for the development of metastases, while alterations in *GNAQ/GNA11* (G protein alpha subunits) are driver mutations with high diagnostic but lesser prognostic value (3, 4).

Treatment of primary UM usually consists of various non-surgical approaches for local tumor control preventing enucleation (e. g. external beam radiation or brachytherapy) and frequently preserving vision. In other cases, a surgical approach with enucleation of the affected eye can become necessary (5).

There are several treatment options for metastatic UM, depending on its pattern of metastatic spread, speed of progression and molecular profile. No guidelines are currently available. Liver-directed treatments are usually the first treatment of choice (if no other metastases are present or at least, liver metastases are prognosis-leading) and include hepatic resection, chemosaturation/isolated hepatic perfusion (IHP) or hepatic arterial chemoembolization. Recent data suggest that IHP has a high response rate and an overall survival benefit of about 14 months (6).

Immune checkpoint inhibitors (ICI) such as ipilimumab (anti-CTLA4) and nivolumab (anti-PD-1) improved prognosis of cutaneous melanoma. In the 6.5-year outcome of the CheckMate 067 trial the median overall survival of previously untreated patients with stage III (unresectable) or stage IV melanoma was 72.1 months in the group that received the combined regimen with ipilimumab (3 mg/kg) and nivolumab (1 mg/kg) once every three weeks for four doses (7).

There have been several prospective trials and retrospective analyses investigating ICI in patients with metastatic uveal melanoma. Ultimately, ICI have shown disappointing results compared to those achieved in patients with cutaneous melanoma. In contrast to cutaneous melanoma, combined ICI has limited impact in metastatic UM. Median progression-free survival (mPFS) ranges from 3-5.5 months and median overall survival (mOS) ranges from 12.7-19.1 months in phase 2 clinical trials (8, 9).

The underlying mechanisms of this ICI resistance are complex and not yet fully understood. Studies have shown a correlation between efficacy of ICI and a high tumor mutational burden which is common for cutaneous malignant tumors such as cutaneous melanoma or cutaneous squamous cell carcinoma. In contrast to this, uveal melanoma shows an exceptionally low tumor mutational burden (18 vs. 1.1 mutations per Mb) (10, 11). Accordingly, PD-L1 expression rates are substantially lower in metastatic uveal melanoma than metastatic cutaneous melanoma. This combined lack of neoantigens and PD-L1 expression suggests immune evasion of tumor cells (12). The eye itself is a so-called immune privileged site. It is shielded from the classical immune response (in particular the release of inflammatory mediators and macrophages) which could have dramatic consequences on tissue with limited regenerative capacity. The poor responsiveness to ICI of metastatic UM suggests that this shielded immunological environment is also recreated in metastatic tissue (13, 14).

In April 2022, tebentafusp (a bispecific gp100 peptide-HLA-directed CD3 T cell engager) has been approved in the European Union as systemic therapy for HLA-A*02:01 positive patients with metastatic uveal melanoma. The estimated median overall survival was 21.7 months (18.6-28.6), median progression-free survival was 3.3 months (3-5) (15). However, the median duration of response was rather short. Currently, there are several retrospective studies investigating therapy sequences in uveal melanoma. First results revealed a tendency towards a better overall survival in patients who progressed on tebentafusp and then received ICI compared to patients who progressed on ICI und were subsequently treated with tebentafusp. In the subsequent evaluation of a randomized phase III trial of metastatic uveal melanoma with first-line either tebentafusp or investigator’s choice, patients with post-progression ICI appeared to have a better overall survival when they had been treated with tebentafusp before compared to ICI before tebentafusp (16).

In a small, single center retrospective cohort study comparing retrospectively 10 patients in each group treated by tebentafusp followed by ICI and vice versa, there was a significant survival benefit for the patients receiving ICI after progressive disease under tebentafusp (17).

## 2 Case presentation

A 78-year-old male patient was clinically diagnosed with uveal melanoma of the left eye in December 2019. The initial staging (cMRI, liver MRI, full body CT) remained tumor-free without any metastases. A skin examination was unsuspicious. In the following, an enucleation of the left eye was performed confirming the diagnosis pathologically as uveal melanoma with invasion of the sclera and an emissary vessel. The patient had no relevant concomitant diseases.

Within the follow-up the patient received eye ophthalmological controls every 3 months and a liver MRI every 6 months. First liver metastases were detected by liver MRI of July 2021, when new suspect hepatic lesions in segments VI and VIII were noted. As there was no sign of extrahepatic metastases, a liver-specific procedure was performed – first, a transarterial chemoembolization of the two metastases in segment VI and segment VIII and after notion of further hepatic progression in September 2021, a chemosaturation. A new hepatic lesion in segment VI was treated with a second transarterial chemoembolization in January 2022.

In March 2022 new lung metastases, soft tissue metastases, and size-progressive liver metastases were detected. At this time, an HLA analysis had already been conducted and had confirmed HLA-A*02:01 positivity in the patient. As tebentafusp had recently been approved for HLA-A*02:01 positive patients with metastatic uveal melanoma, the patient received this treatment as one of the first patients outside of studies or early access program (EAP) at the University Hospital of Tübingen in April 2022. After the first cycle (20 µg) he experienced severe side effects with, fever, acute renal injury and elevation of CRP and liver enzymes. These side effects were treated symptomatically and also the uric acid was lowered with rasburicase as tumor lysis syndrome was suspected. The patient recovered quickly, but developed a cytokine release syndrome at the third cycle (30 µg) which was treated with tocilizumab. In the following, the symptoms decreased with each cycle; the full dose of tebentafusp was administered at the fifth cycle (68 µg). In total, the patient received 11 cycles of tebentafusp until June 2022.

**Figure 1:**
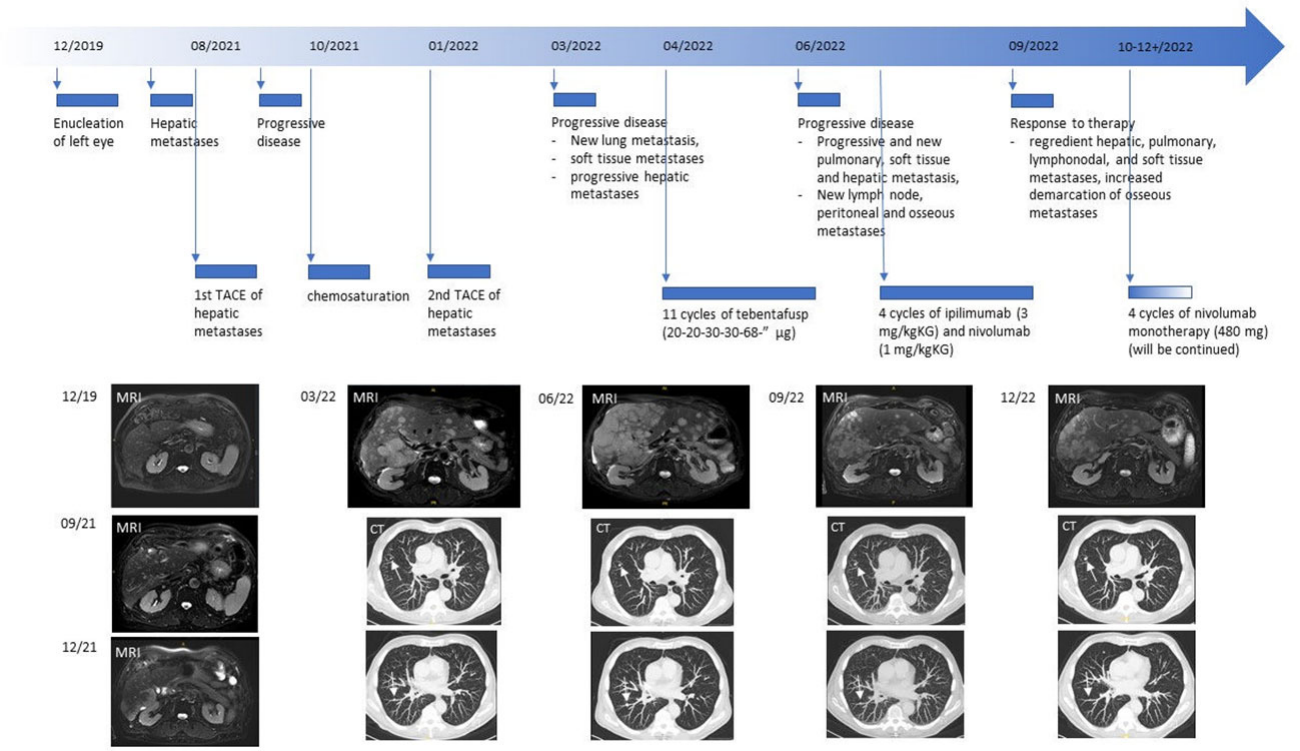
Course of the disease.

The next full body staging was conducted in June 2022 which showed progressive pulmonary, hepatic and soft tissue metastases and also new osteolytic metastatic lesions. Consequently, the treatment with tebentafusp was discontinued and combined ICI was started 11 days after the last administration of tebentafusp. The patient received 4 cycles of ipilimumab (3 mg per kilogram body weight) and nivolumab (1 mg per kilogram body weight) which he tolerated without any side effects. The following staging of September 2022 showed a very good response with considerably reduced hepatic, pulmonary, lymphonodal and soft tissue metastases and increased demarcation of osseous metastases. In October 2022, the treatment was continued as recommended with nivolumab as monotherapy (480 mg, q28). Before the start of the combined immunotherapy, LDH levels had been elevated up to 680 U/l. After 4 cycles of immunotherapy, the LDH levels dropped down to 315 U/l and further decreased at the time of the second follow-up at the end of December 2022 with almost normal LDH levels (269 U/l). The staging revealed stable findings (**Figure 1**).

In addition to the HLA analysis which was performed in December 2021, Next-Generation Sequencing (NGS) was conducted after progression on tebentafusp and before starting combined ICI in June 2022. Two somatic changes were found in the *BAP1* gene, one being a frameshift mutation (c.908_918del, p.Ala303GlyfsTer91), another being a heterozygotic deletion in chromosome 3 (chr3; p21.1p21.31). Another somatic change was a missense mutation in the *GNA11* gene (c.626A>T, p.Gln209Leu). As a consequence, the molecular tumor board suggested an off-label use of the EZH2 inhibitior (Tazemetostat) as future therapy option in case of progression under ICI.

**Figure 2:**
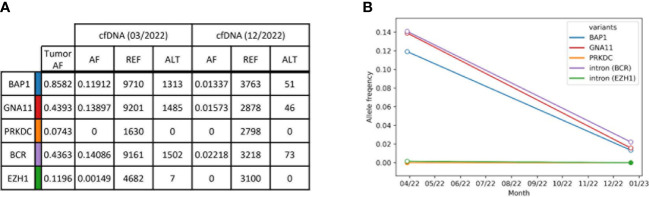
Summary of tumor variants monitored in plasma cfDNA. **(A)** Selected tumor variants detected by tumor normal sequencing with a comprehensive sequencing panel. Resulting variants were selected for a smaller tumor-specific hybridization-based enrichment panel and sequenced to an ultra-high depth. Allele frequencies (AF) were obtained from reference reads (REF, GRCh38) and alternative reads (ALT). Reads without duplicates were excluded during sequencing error correction. **(B)** ctDNA kinetics of selected tumor variants. Each line represents one variant. The allele frequencies are plotted over time and refer to reads with at least one duplicate. White circles indicate that the variant was found with a p-value < 0.05.

Based on the results from tumor normal sequencing, a tumor-specific enrichment panel was designed for hybridization capture NGS. Targeted ultra-deep sequencing was conducted with plasma cell-free DNA (cfDNA) obtained from peripheral blood. A total of five variants was analyzed at two different time points of therapy (**Figure 2A**, **Supplementary Table 1**). The first time point was baseline before the initiation of tebentafup, while the second time point fell into the period of response under combined ICI with regredient metastases. Unfortunately, no cfDNA sample was taken at the time point of progressive disease under tebentafusp. Four out of five tumor variants were detected at the first time point. In accordance with the clinical findings and imaging results, the allele frequencies were markedly reduced at time point 2 (**Figure 2B**). The only variant (*PRKDC*) not found in any of the cfDNA samples already displayed the lowest allele frequency (AF) in the tumor.

## 3 Discussion

There are high expectations regarding the new treatment option for advanced HLA-A*02:01 UM with tebentafusp. Nevertheless, the optimal sequence of tebentafusp and combined ICI is unknown (17, 18). We have seen an unexpectedly rapid and profound response in our patient to combined ICI after previous tebentafusp therapy and we would like to outline possible reasons that might argue for the use of combined ICI in case of progression to tebentafusp.

It is known that ICI is less effective with liver metastases (7, 19, 20). Median progression-free survival was markedly reduced for melanoma patients of the checkmate 067 study in case of baseline liver metastases (4.4 months; 2.8-11.5) compared to patients without baseline liver metastases (18.1 months; 10.7-42.7). Likewise, overall survival was found to be markedly worse (28.2 months; 15.2-71.9) when baseline liver metastases were present compared to patients without liver metastases at the beginning of ICI (NR; 50.7- NR) (7). Considering a 59% rate of grade 3-4 immune-mediated adverse events with a lower response rate than in cutaneous melanoma, the first-line use of combined ICI in metastatic uveal melanoma should be considered restrictive.

In an evaluation concerning the effect of post-progression treatment on the outcome of patients that had been included in the phase III trial of first-line tebentafusp or investigator’s choice, patients with ICI after progression on tebentafusp tended to have improved survival (16).

Furthermore, a subgroup of patients from the first phase I trial, who were progressive on ICIs and then also progressive on tebentafusp, in some cases responded to a re-challenge of ICIs after all (21).

It is known that the application of tebentafusp results in an increase of T cells in the tumor microenvironment, as well as an increase of IFNγ, CXCL9, CXCL10 and CXCL1. Adverse effects such as skin rash or pruritus are probably due to the interaction of T cells with melanocytes expressing gp100 in the skin. Patients that experienced rash within the first week of tebentafusp treatment had a significant better 1-year overall survival rate (83%) compared to patients without rash (59%). High CXCL10 expression is known to be a predictive marker for treatment response to ICI. The predictive power of CXCL10 was even better than that of PD-1/PD-L1 (22, 23).

Considering the above-mentioned effect of tebentafusp on the tumor microenvironment, it might be reasonable to use ICI in metastatic UV primarily after previous treatment with tebentafusp, or at least to try it again after previous ICI progression after intermediate tebentafusp application.

We learned from Tumeh et al. that in cutaneous melanoma, the response to anti-PD-1 antibodies is based on the presence of tumor-infiltrating lymphocytes with a high proportion of CD8+ T cells (24). In terms of response, this could translate to UM, such that the aforementioned increase in T cells in the melanoma environment due to tebentafusp-induced recruitment and activation of T cells in the vicinity of gp100-peptide HLA-presenting melanoma cells becomes crucial. In addition, *in vitro* studies have shown that tebentafusp enhanced epitope spreading, whereby tumor-associated antigens released by apoptotic tumor cells are captured and displayed by dendritic cells, which then induce T cells to lyse additional melanoma cells (25). It is further known that chronic activation induces an exhausted phenotype in T cells characterized by the expression of exhaustion markers and inhibitory checkpoints such as PD-1 (26). This suggests a sequential therapeutic regimen with tebentafusp followed by immune checkpoint inhibition as a logical next step. With regard to the cycle of cancer immunity (27), this can be interpreted as enhanced recruitment and infiltration of T cells into the tumor (steps 4 and 5 of the cycle of cancer immunity) by tebentafusp, including T cells that recognize cancer cells (step 6 of the cycle), and enhanced T cell-mediated killing of melanoma cells by anti-PD-1-based ICI (step 7 of the cycle). In addition, the combination with anti-CTLA-4 could promote priming and activation of additional T cells (step 3 of the cycle).

Our case report underlines this regime with an excellent response to ICI after previous progression on tebentafusp. Furthermore, we were able to detect selected driver and passenger mutations in the personalized liquid biopsy, that could be performed baseline before initiation of tebentafusp and afterward under ICI. With this approach we were able to detect the lowly-abundant *EZH1* variant with an AF of 0.15%, and sensitivity was only limited by sequencing depth. In principle, circulating tumor DNA (ctDNA) monitoring can also be used for response evaluation. It was shown that in patients who were treated with tebentafusp, ctDNA reduction correlated with survival but not necessarily RECIST response. However, repeated ctDNA evaluations are not yet part of the daily routine of clinical care. In our case the ctDNA result at the time point of progression under tebentafusp would have been interesting. However, we have here only the two points in time before the start of therapy with tebentafusp and after 4 cycles of combined immunotherapy.

The main limitation of our case is that only one radiological diagnosis was made before switching to combined ICI due to the multifocal and extensive progression. In the phase III study, treatment beyond progression was allowed under certain circumstances (15).

Based on this, our patient would have been allowed to receive another round of treatment with tebentafusp under study conditions for at least 4 weeks. However, since the progression was severe and there were many new metastases, there probably would not have been enough time to wait for another progress and only then to change the treatment regime. We suppose that the immediate switch to combined ICI and the tumor microenvironment being optimized by tebentafusp that probably enriched relevant chemokines such as CXCL10 in the tumor, has been essential for the excellent response to ICI.

Patients receiving tebentafusp under study conditions beyond progression were required to permanently discontinue study treatment if a further progression occurred, defined as any of the following events occurring at least 4 weeks after the initial PD assessment:

- An additional increase in tumor burden of ≥ 20%.
- Progressive disease of non-target lesions.
- New non-measurable lesions (15).

For the future, we suggest to start first-line tebentafusp in case of non-resectable metastases of UM. If there are progressive findings in the first follow-up, a continuation with tebentafusp should be considered. In case of further progression of metastases, treatment with combined ICI might be an option. Further studies are necessary to compare prospectively the optimal treatment sequence of tebentafusp and ICI, added by ctDNA monitoring by NGS.

## Data availability statement

The original contributions presented in the study are included in the article/**Supplementary Material**. Further inquiries can be directed to the corresponding author.

## Ethics statement

Written informed consent was obtained from the patient for the publication of any potentially identifiable images or data included in this article. The study was performed in accordance with the Declaration of Helsinki, Good Clinical Practice and applicable regulatory requirements.

## Author contributions

Conception and design: SR, AF. Acquisition of data: SR, CS, AF. Analysis and interpretation of data: SR, CS, TS, LF, AF. Writing, review, revision of the manuscript: all authors. Study supervision: AF, LF. All authors contributed to the article and approved the submitted version.

## Supplementary material

Supplementary Table 1 Coding information of tumor variants selected for monitoring in cfDNA. GRCh38 was used as reference genome.
