# Supplementary material for: Successful treatment of metastatic uveal melanoma with ipilimumab and nivolumab after severe progression under tebentafusp: a case report
Source: Front Oncol. 2023 May 3;13:1167791. doi: 10.3389/fonc.2023.1167791 (PMC10189013; doi:10.3389/fonc.2023.1167791)
Supplement: Supplementary Table 1 — Coding information of tumor variants selected for monitoring in cfDNA. GRCh38 was used as reference genome. [file Table_1.docx]

| Gene | Reference | Observed | cDNA | Protein | Variant type |
| --- | --- | --- | --- | --- | --- |
| BAP1 | CTCAGAGGCTG | - | c.908_918del | p.Ala303GlyfsTer91 | Frameshift |
| PRKDC | G | T | c.3848-4C>A | n/a | Splice region & intron |
| EZH1 | A | C | c.997-59T>G | n/a | Intron |
| GNA11 | A | T | c.626A>T | p.Gln209Leu | Missense |
| BCR | A | G | c.1279+10030A>G | n/a | Intron |
